# Overview of glottic laryngeal cancer treatment recommendation changes in the NCCN guidelines from 2011 to 2022

**DOI:** 10.1002/cnr2.1837

**Published:** 2023-06-07

**Authors:** Lady Paola Aristizabal Arboleda, Aline Borburema Neves, Hugo Fontan Kohler, José Guilherme Vartanian, Letícia Miliano Candelária, Matheus Ferraz Borges, Gisele Aparecida Fernandes, Genival Barbosa de Carvalho, Luiz Paulo Kowalski, Paul Brennan, Alan Roger Santos‐Silva, Maria Paula Curado

**Affiliations:** ^1^ Graduate Program A.C. Camargo Cancer Center São Paulo Brazil; ^2^ Head and Neck Surgery and Otorhinolaryngology Department A.C. Camargo Cancer Center São Paulo Brazil; ^3^ Group of Epidemiology and Statistics on Cancer A.C. Camargo Cancer Center São Paulo SP Brazil; ^4^ Head and Neck Surgery Department, Faculdade de Medicina Universidade de São Paulo São Paulo Brazil; ^5^ International Agency for Research on Cancer Genomic Epidemiology Branch Lyon France; ^6^ Oral Diagnosis Department, Piracicaba Dental School University of Campinas Campinas Brazil

**Keywords:** glottic cancer, laryngeal cancer, NCCN guidelines, review, treatment

## Abstract

**Background:**

The treatment of glottic cancer remains challenging, especially with regard to morbidity reduction and larynx preservation rates. The National Comprehensive Cancer Network (NCCN) has published guidelines to aid decision‐making about this treatment according to the tumor site, clinical stage, and patient medical status.

**Aim:**

The present review was conducted to identify changes in the NCCN guidelines for glottic cancer treatment made between 2011 and 2022 and to describe the published evidence concerning glottic cancer treatment and oncological outcomes in the same time period.

**Methods and Results:**

Clinical practice guidelines for head and neck cancer published from 2011 up to 2022 were obtained from the NCCN website (www.NCCN.org). Data on glottic cancer treatment recommendations were extracted, and descriptive analysis was performed. In addition, a review of literature registered in the PubMed database was performed to obtain data on glottic cancer management protocols and treatment outcomes from randomized controlled trials, systematic reviews, and meta‐analyses published from 2011 to 2022. In total, 24 NCCN guidelines and updates and 68 relevant studies included in the PubMed database were identified. The main guideline changes made pertained to surgical and systemic therapies, the consideration of adverse features, and new options for the treatment of metastatic disease at initial presentation. Early‐stage glottic cancer received the most research attention, with transoral endoscopic laser surgery and radiotherapy assessed and compared as the main treatment modalities. Reported associations between treatment types and survival rates for this stage of glottic cancer appear to be similar, but functional outcomes can be highly compromised.

**Conclusion:**

NCCN panel members provide updated recommendations based on currently accepted treatment approaches for glottic cancer, constantly reviewing new surgical and non‐surgical techniques. The guidelines support decision‐making about glottic cancer treatment that should be individualized and prioritize patients' quality of life, functionality, and preferences.

## 1 INTRODUCTION

The incidence and mortality rates of laryngeal cancer are high worldwide, with an estimated 184 615 new cases diagnosed and 99 840 deaths occurring in 2020.^1^ Laryngeal cancer affects the glottis in 70% of cases, but can also affect the supraglottis and subglottis. Squamous cell carcinoma (SCC) is its main histopathological type.^2^, ^3^

The application of the American Joint Committee on Cancer's TNM staging system for glottic cancer is crucial for the selection of the most appropriate treatment option.^4^ Glottic SCC can be treated with the individual or combined application of surgery, radiotherapy (RT), and chemotherapy. Surgery and RT are the two options for early‐stage (T1–T2) glottic cancer and, because nodal disease at these stages is rare, survival rates are high (90% for patients receiving RT, 93% for those undergoing transoral microsurgery).^5^, ^6^ Although treatment choices depend on institutional human and technological resources and patient factors, transoral microsurgery with preservation of the larynx yields better functional and quality of life (QoL) outcomes.^5^, ^7^ Traditional oncological protocols for advanced (stage III–IV) glottic cancer include techniques such as total laryngectomy and postoperative RT or non‐surgical therapy (chemotherapy). Overall and disease‐specific survival rates have been better following total laryngectomy than after non‐surgical therapy,^8^ but current evidence shows that induction chemotherapy followed by RT yields superior clinical outcomes due to decreased morbidity and organ preservation.^7^, ^9^

The National Comprehensive Cancer Network (NCCN) has developed clinical practice guidelines for the screening, prevention, diagnosis, treatment, and follow‐up of different types of cancer, including head and neck cancers. Recommendations in these guidelines are updated frequently following the critical review of newly published high‐level evidence and the establishment of consensus by multidisciplinary panels of experts, thereby providing appropriate orientation for decision making about oncological care.^10^, ^11^ Head and neck cancer treatment protocol recommendations may differ according to country‐specific conditions, but the overall standardization of recommendations underpinned by high‐level scientific evidence and improvements in clinical outcomes is needed to reduce discrepancies in patients' clinical responses.^12^

Clinical scenarios for decision making about glottic cancer treatment, including patient preferences and institutional conditions, often reveal challenges. The possibility of the occurrence of morbidities associated with essential functions, such as the loss of the natural voice, breathing, and airway protection during swallowing, should be evaluated carefully.^13^ Divergent survival and clinical outcomes of various oncological protocols have been reported, creating controversy about treatment choices, especially regarding organ preservation in patients with advanced (T3–T4) glottic cancer and well‐documented reductions in survival rates.^8^, ^14^, ^15^, ^16^, ^17^ Thus, the main goal of the present review was to describe the main changes made to the NCCN guidelines for glottic cancer treatment published between 2011 and 2022, as these guidelines serve as the reference in many institutions treating head and neck cancer worldwide. Secondary objectives were to describe the main features of references used in NCCN guideline development and relevant PubMed‐registered literature from 2011 to 2022, to provide an overview of published evidence for glottic cancer treatment types and oncological outcomes.

## 2 METHODS

### 2.1 Search strategy

The NCCN Clinical Practice Guidelines (NCCN Guidelines®) for Head and Neck Cancers published between 2011 and 2022 in the *Journal of the National Comprehensive Cancer Network* were obtained by a search using keywords such as “guidelines,” “head and neck,” “larynx,” and “glottis.” Archived guidelines that were not available in the journal were requested via the NCCN website (www.NCCN.org).

A systematic search of the PubMed database was performed to identify reports on randomized controlled trials (RCTs), systematic reviews (SRs), and meta‐analyses (MAs) published between 2011 and 2022 that provided data on functional (voice, swallowing, QoL) and survival outcomes for glottic SCC by treatment modality (Table A1). Exclusion criteria were applied for: (1) studies other than treatment of glottic cancer; (2) glottic cancer treatment focusing on recurrence or rehabilitation; (3) studies without functional (voice, swallowing, QoL) and survival outcomes; (4) non‐SCC on the glottic larynx or SCC involving other than glottis; and (5) observational studies, case reports, series reports, narrative literature reviews, guidelines, and letters to the editor.

### 2.2 Data extraction

Data recording and descriptive analysis were performed using Excel software (Microsoft Corporation, Redmond, WA, USA). The following information was extracted from the NCCN guidelines: year, version, updates, characteristics of the references on glottic cancer treatment used (authors, publication year, country, study design), and recommended treatment algorithms for all clinical stages of glottic cancer. For the publications obtained by PubMed database search, the authors, publication year, country, study design, clinical stage, outcomes, treatment modality, and main results were extracted.

## 3 RESULTS

In total, 24 updates of the NCCN guidelines were published between 2011 and 2022, with the number published varying among years. They contained mainly workup recommendations; descriptions of the principles of surgery, RT, and systemic therapy (ST); wording on clinical stages; primary and neck treatment pathways; descriptions of adverse features; and categories of evidence and preference. The largest numbers of changes occurred between 2013 and 2014 and between 2018 and 2019 (Table 1).

**TABLE 1:** Major changes in NCCN guideline recommendations for glottic cancer treatment, 2011–2022.

Type of change	Early		Advanced			Very advanced	
	Carcinoma in situ	Amenable to larynx‐preserving (conservation) surgery (T1–T2, N0 or select T3, N0)	T3 requiring (amenable to) total laryngectomy (N0–1)	T3 requiring (amenable to) total laryngectomy (N2–3)	T4a disease	T4b, N0–3 or unresectable nodal disease or unfit for surgery	Metastatic (M1) disease at initial presentation
Clinical staging	‐	2012, 2013, 2015, 2019	2013	2013	‐	2019	2019
Primary and neck treatment							
Treatment pathway options	2012	‐	2013, 2014	‐	‐	2013, 2020, 2021	2013, 2021, 2022
Surgical pathway recommendations	‐	2014, 2015, 2016	2011, 2013, 2015, 2018, 2019	2013, 2016, 2018	2012, 2018, 2019, 2020	‐	‐
Category of evidence and preference	2014	‐	2019	2014	2014	2019	‐
Response after induction chemotherapy	‐	‐	‐	2014, 2017, 2018	2014, 2017, 2018	‐	‐
Adjuvant treatment							
No adverse/adverse features	‐	2012, 2013, 2014	2017	‐	2018	‐	‐
Pathway recommendations	‐	‐	‐	‐	2012, 2017	‐	‐
General							
Workup (2012, 2019, 2022), principles of surgery (2016, 2018, 2019, 2021, 2022), principles of radiation therapy (2011, 2014, 2019), principles of systemic therapy (2012, 2013, 2018, 2019, 2021, 2022).							

The main changes to workup recommendations pertain to the need for radiological imaging according to the clinical stage of glottic cancer. Currently, chest computed tomography (CT, with or without contrast), fluorodeoxyglucose positron emission tomography/CT, and pulmonary function evaluation are recommended for conservation surgery candidates. Diagnosis still requires full history taking and physical examination, biopsy of the primary tumor site or fine needle aspiration of the neck, CT with contrast and thin angled cuts through larynx, and/or magnetic resonance imaging of the primary site and neck.

The main changes in glottic cancer treatment pathways are described by clinical stage in Table 2. For carcinoma in situ (Tis), clinical trials were removed as an option for treatment delivery, and endoscopic resection was deemed the preferred treatment. For stage T1–T2 and select stage T3 N0 cases, neck dissection (as indicated) was recommended and a new pathway based on adverse features was provided. For stage T3 N0–1 cases, two new pathways for primary and neck treatment (involving induction chemotherapy and clinical trials) were provided and the surgical recommendations were modified to favor pretracheal and ipsilateral paratracheal lymph‐node dissection. For patients receiving concurrent ST and RT, the adjuvant therapy pathway was removed. For stage T3 N2–3 cases, pretracheal and ipsilateral paratracheal lymph‐node dissection was recommended. The evidence category for induction chemotherapy was changed from 3 to 2A, and clinical trials have been recommended as an option for treatment delivery since 2015. Treatment pathways according to induction chemotherapy responses were modified significantly, and the evidence categories for RT in cases of complete response and ST/RT in cases of partial response were changed to 1 and 2B, respectively. For T4a N0–3 cases, the surgical and adjuvant treatment recommendations were changed considerably, with pretracheal and ipsilateral paratracheal lymph‐node dissection recommended and postoperative adjuvant treatment pathways provided according to adverse features. The category of evidence for induction chemotherapy for patients declining surgery was changed from 2B to 2A. For T4b N0–3 cases, recommendations based on patients' performance status (PS) were changed, and the evidence categories for concurrent ST and RT and induction ST followed by RT or concurrent ST and RT for patients with PSs of 0–1 were changed from 1 and 3, respectively, to 2A. Palliative RT was added as an option for patients with PSs of 3. For glottic cancer that is metastatic (M1) at initial presentation, a new algorithm was provided in 2015.

**TABLE 2:** NCCN guideline recommendations for glottic cancer treatment, 2011–2022.

Stage	Primary treatment	Years	Neck treatment	Years	Adjuvant treatment	Years
Tis	1. ER	2011–2022	‐	‐	‐	‐
	Preferred	2014–2022				
	2. RT	2011–2022				
	3 Clinical trial	2011–2012				
T1–2 N0, selected T3	1. RT	2011–2022			Follow‐up:	2014–2022
	2. Partial laryngectomy with ER or open resection (as indicated)	2011–2022	Neck dissection as indicated	2015–2022	No adverse feature: observation	2014–2022
					Adverse features: Extranodal extension: ST/RT (category 1) Positive margins: re‐resection or RT Other risk features: RT	2014–2022
T3 N0–1	1. Concurrent ST/RT or, if not candidate, RT	2011–2022			Follow‐up neck evaluation:	2017–2022
	2. Laryngectomy	2011–2022	N0: ipsilateral thyroidectomy (as indicated), pretracheal and ipsilateral paratracheal lymph‐node dissection N1: also ipsilateral or bilateral neck dissection	2018–2022	No adverse feature: observation Adverse features: Extranodal extension and/or positive margins: ST/RT (category 1) Other risk features: RT or consider ST/RT	2011–2022
	3. Induction chemotherapy	2014–2022	CT or MRI (with contrast) of primary site and neck	2014–2022	CR: definitive RT (category 1) PR: RT (category 1) or ST/RT (category 2B) <PR: laryngectomy or unresectable nodal disease	2014–2022
	4. Clinical trials	2013–2022				
T3 N2–3	1. Concurrent ST/RT	2011–2022			Follow‐up neck evaluation:	2018–2022
	2. Laryngectomy	2011–2022	Thyroidectomy, ipsilateral or bilateral neck dissection, pretracheal and ipsilateral paratracheal lymph‐node dissection	2018–2022	No adverse feature: follow‐up Adverse features: Extranodal extension and/or positive margins: ST/RT (category 1) Other risk features: RT or consider ST/RT	2011–2022
	3. Induction chemotherapy	2011–2022	CT or MRI (with contrast) of primary site and neck	2018–2022	CR: definitive RT (category 1) PR: RT (category 1) or ST/RT (category 2B) <PR: laryngectomy or unresectable nodal disease	2017–2022
	4. Clinical trials	2015–2022				
T4a, N0–3	1. Surgery: Total laryngectomy 2. For selected T4a patients who decline surgery: Consider concurrent ST/RT Clinical trial for function‐preserving surgical or nonsurgical management Induction chemotherapy	2011–2022 2011–2022 2015–2022	N0: thyroidectomy ± unilateral or bilateral neck dissection, pretracheal and ipsilateral paratracheal lymph‐node dissection N1: thyroidectomy, ipsilateral or bilateral neck dissection, pretracheal and ipsilateral paratracheal lymph‐node dissection N2–3: total laryngectomy with thyroidectomy, ipsilateral or bilateral neck dissection, pretracheal and ipsilateral paratracheal lymph‐node dissection	2018–2022	No adverse features: follow‐up Adverse features: Extranodal extension and/or positive margins: ST/RT (category 1) Other risk features: RT or consider ST/RT	2018–2022
T4b any N, unresectable nodal disease or unfit for surgery	1. Clinical trial preferred 2. Standard therapy PS 0–1: concurrent ST/RT or induction ST + RT or ST/RT PS 2: RT or concurrent ST/RT PS 3: palliative RT, single‐agent ST, or best supportive care	2011–2022 2011–2022 2021–2022	Individual decision Tumor board discussion			
Metastatic (M1) disease at initial presentation	1. Clinical trial preferred 2. Consider locoregional treatment based on primary site algorithms 3. Standard ST PS 0–1: combination ST, single‐agent ST, surgery or RT, ST/RT for selected cases with limited metastases, or best supportive care PS 2: single‐agent ST, best supportive care, palliative RT, or palliative surgery PS 3: best supportive care, palliative RT, or palliative surgery	2015–2022 2015–2022 2021–2022	Individual decision Tumor board discussion		ST, clinical trial preferred or palliative RT or best supportive care Best supportive care, alternate single‐agent ST, or palliative RT	2022

Abbreviations: CR, complete response; CT, computed tomography; ER, endoscopic resection; MRI, magnetic resonance imaging; PR, partial response; PS, performance status; RT, radiation therapy; ST, systemic therapy.

**FIGURE 1:**
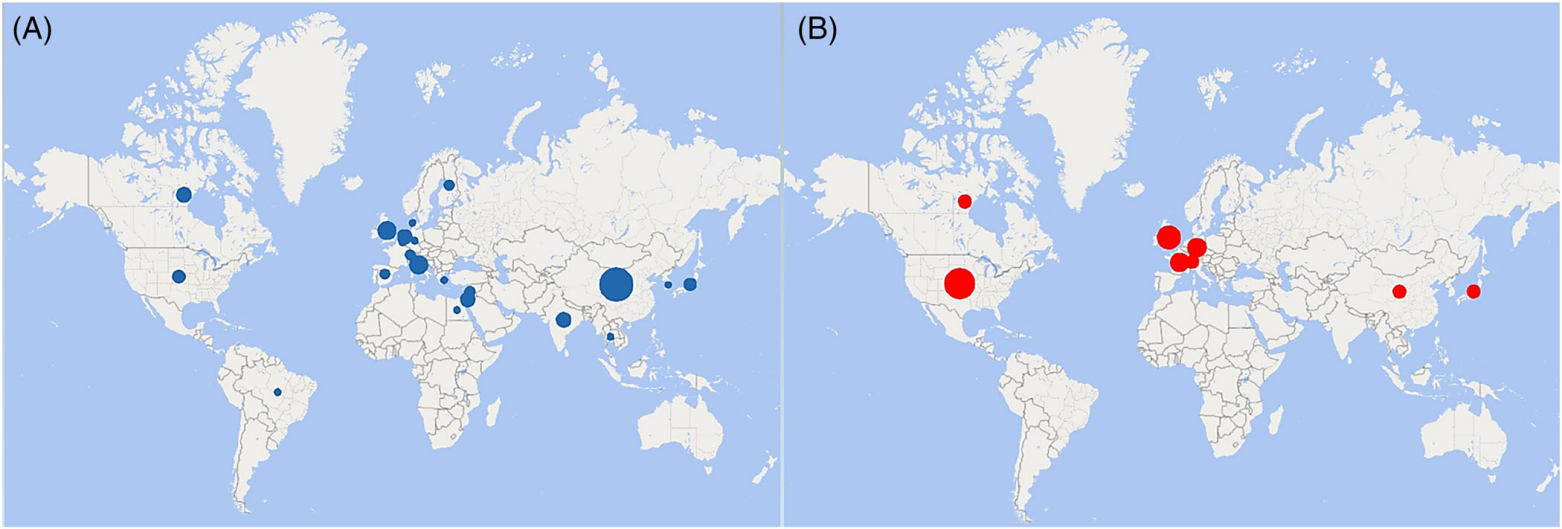
Overview of countries in which researchers have contributed to the scientific literature on laryngeal glottic cancer treatment identified by PubMed search (A) and cited in the NCCN guidelines (B), 2011–2022.

A total of 19 studies [8 RCTs,^9^, ^18^, ^19^, ^20^, ^21^, ^22^, ^23^, ^24^ 4 observational studies,^25^, ^26^, ^27^, ^28^ 3 SRs,^29^, ^30^, ^31^ 2 cohort studies,^32^, ^33^ 1 narrative review,^34^ and 1 MA^35^] contributed to the updating of the NCCN guidelines between 2011 and 2022. The SRs and MA were included as references in the guidelines in recent years. The studies were performed in the United States,^9^, ^19^, ^20^, ^21^, ^33^, ^34^ United Kingdom,^26^, ^29^, ^30^ Switzerland,^18^, ^25^, ^28^ Germany,^27^, ^32^ France,^22^, ^23^ China,^35^ Canada,^31^ and Japan^24^ (Figure 1).

**FIGURE 2:**
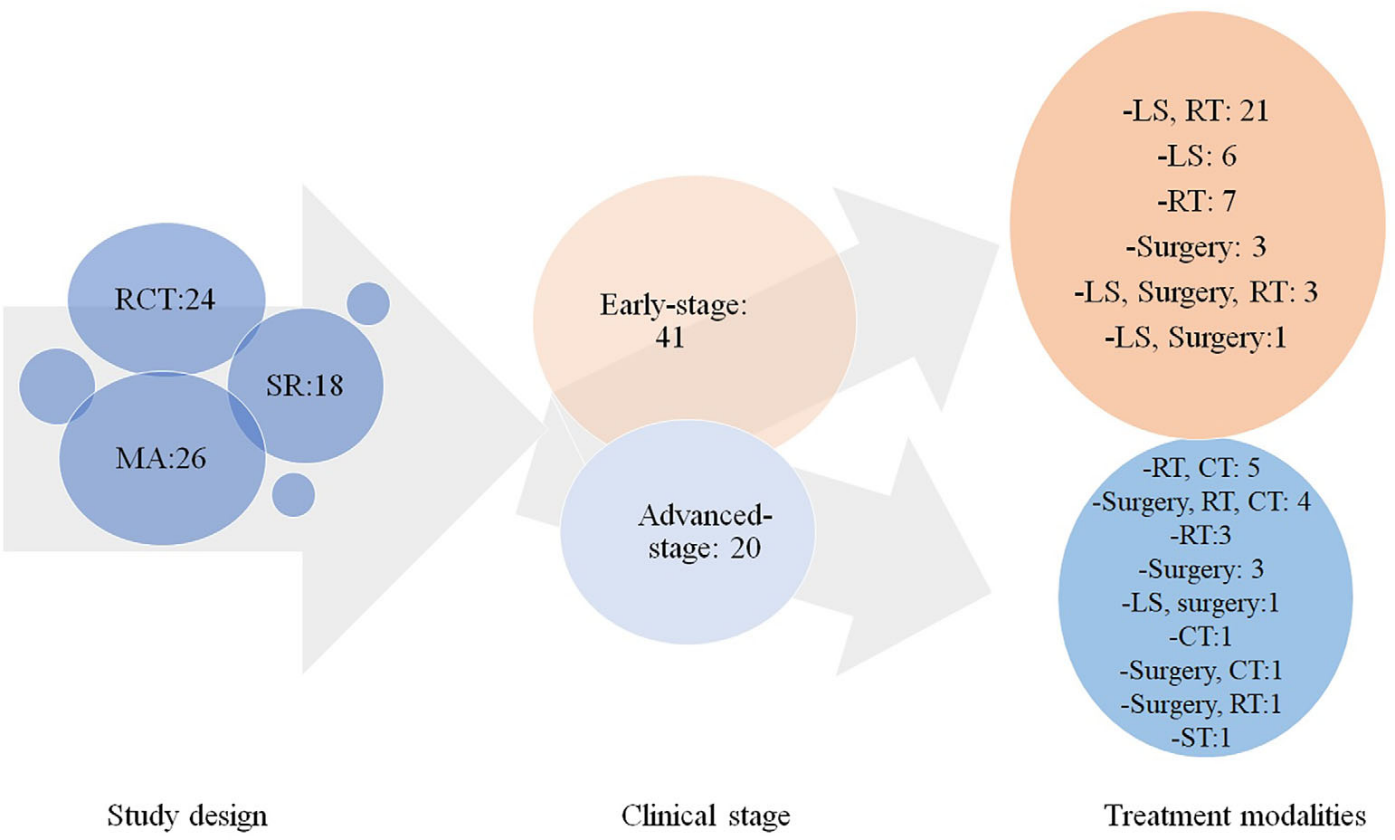
Characteristics of studies registered in the PubMed database (2011–2022). CT, chemotherapy; MA, meta‐analysis; LS, laser surgery; RT, radiotherapy; RCT, randomized controlled trial; ST, systemic therapy; SR, systematic review.

The PubMed database search yielded a total of 260 studies (Table A1), of which 68 (26 MAs, 24 RCTs, and 18 SRs) fulfilled the selection criteria.^9^, ^13^, ^14^, ^15^, ^29^, ^30^, ^31^, ^33^, ^35^, ^36^, ^37^, ^38^, ^39^, ^40^, ^41^, ^42^, ^43^, ^44^, ^45^, ^46^, ^47^, ^48^, ^49^, ^50^, ^51^, ^52^, ^53^, ^54^, ^55^, ^56^, ^57^, ^58^, ^59^, ^60^, ^61^, ^62^, ^63^, ^64^, ^65^, ^66^, ^67^, ^68^, ^69^, ^70^, ^71^, ^72^, ^73^, ^74^, ^75^, ^76^, ^77^, ^78^, ^79^, ^80^, ^81^, ^82^, ^83^, ^84^, ^85^, ^86^, ^87^, ^88^, ^89^, ^90^, ^91^, ^92^, ^93^, ^94^ The countries in which the largest numbers of studies were performed were China (*n* = 18), the United Kingdom (*n* = 6), and Italy (*n* = 5; Figure 1). The main characteristics of the included publications are summarized in Table 3. The treatment of early‐stage glottic cancer, was examined in 44 studies,^29^, ^30^, ^31^, ^35^, ^36^, ^37^, ^38^, ^40^, ^41^, ^45^, ^46^, ^48^, ^50^, ^51^, ^52^, ^53^, ^54^, ^55^, ^56^, ^57^, ^58^, ^59^, ^62^, ^64^, ^65^, ^71^, ^72^, ^74^, ^75^, ^76^, ^77^, ^79^, ^80^, ^81^, ^82^, ^89^, ^90^, ^91^, ^92^, ^93^, ^94^ mainly with transoral laser microsurgery and RT modalities (Figure 2).

**TABLE 3:** Main characteristics of glottic cancer treatment publications registered in the PubMed database, 2011–2022.

Authors, year	Country	Design	Clinical stage	Intervention(s)/treatment(s)	Outcome(s)	Main finding(s)
García‐León et al. 2017^13^	Spain	SR	Advanced laryngeal cancer	Organ preservation (chemotherapy), surgery	QoL	Treatment‐related differences in QoL of patients with advanced laryngeal cancer cannot be established due to insufficient number of studies
Forastiere et al. 2013^9^	USA	RCT	Advanced laryngeal cancer	Induction chemotherapy (cisplatin/fluorouracil) and RT, concomitant cisplatin/RT, RT alone	Laryngectomy‐free survival	Locoregional control and larynx preservation significantly improved with concomitant cisplatin/RT compared with induction chemotherapy and RT alone
Mannelli et al. 2018^14^	Italy	MA	Advanced laryngeal cancer	Transoral laser, open partial laryngectomy	Survival, local control	Both techniques valid conservative surgical options for advanced laryngeal cancer treatment
Li et al. 2019^66^	China	MA	Advanced laryngeal cancer	Postoperative adjuvant RT	Survival	Postoperative adjuvant RT improved survival of patients with surgically managed locally advanced laryngeal cancer
Francis et al. 2014^15^	Lebanon	SR	Advanced laryngeal cancer	Primary total laryngectomy, neck dissection with adjuvant therapy (chemotherapy, RT) when indicated	Survival	High survival rate for primary total laryngectomy for pT4a cases
Khoueir et al. 2015^63^	Lebanon	SR	Advanced laryngeal cancer	Primary total laryngectomy	Survival	Survival better for T4a N0 than T3 N+, especially T3 N2, despite grouping in the same TNM stage IVa
Badwal 2018^39^	India	SR	Advanced laryngeal cancer	Total laryngectomy	Survival	Total laryngectomy remains gold standard for T4a laryngeal cancer management
Singh et al. 2018^83^	India	RCT	Advanced laryngeal cancer	Concurrent chemotherapy/RT	‐	‐
Luo et al. 2015^67^	China	MA	Advanced laryngeal cancer	Total laryngectomy followed by RT, three larynx‐preserving strategies	Survival	Disease‐free survival better for laryngectomy than for chemotherapy and RT, overall survival similar in all groups
Riga et al. 2017^78^	Greece	SR	Advanced laryngeal cancer	Open partial laryngectomy, transoral laser microsurgery, RT with/without chemotherapy	Survival	Survival, organ preservation rates high with partial laryngectomy, microsurgery; preoperative induction chemotherapy compromises overall survival
Tang et al. 2018^87^	China	MA	Advanced laryngeal cancer	Total laryngectomy, nonsurgical organ‐preservation strategies	Survival, local control	Results support total laryngectomy for T4 tumors, no advantage of primary organ preservation, no difference in overall survival for T3 tumors
Ma et al. 2013^69^	China	MA	Advanced laryngeal cancer	Induction chemotherapy	Survival	No difference in overall survival, disease‐free survival, locoregional recurrence with/without induction chemotherapy
Nutting et al. 2021^73^	United Kingdom	RCT	Advanced laryngeal cancer	Dose‐escalated, standard‐dose intensity‐modulated RT	Local control	Dose escalation did not improve locoregional control of laryngeal or hypopharyngeal cancer
Swiecicki et al. 2022^86^	USA	RCT	Advanced laryngeal cancer	Induction chemotherapy (platinum, docetaxel, novel Bcl‐xL inhibitor)	Organ preservation	No difference in laryngeal preservation between one and two cycles
Bonner et al. 2016^42^	Spain, Germany, USA	RCT	Advanced laryngeal cancer	Cetuximab/RT, RT alone	Laryngeal preservation, laryngectomy‐free survival	2‐year laryngeal preservation rates 87.9% with, 85.7% without cetuximab; no difference in overall QoL, feeding tube requirement, or speech
Mesía et al. 2017^70^	Spain	RCT	Advanced laryngeal cancer	Induction chemotherapy (docetaxel, cisplatin, 5‐fluorouracil) followed by bio‐RT	Functional larynx preservation	Survival with functional larynx better than critical value with acceptable toxicity; cetuximab with RT could improve functional larynx preservation in patients with stage III, IVA laryngeal cancer who respond to induction chemotherapy
Stokes et al. 2017^33^	USA	RCT	Advanced laryngeal cancer	Surgical, organ‐preservation modalities (RT, chemotherapy)	Survival	Overall survival better with surgery/adjuvant RT than with concurrent chemotherapy/RT but not different from induction chemotherapy/RT; findings require validation, surgery with adjuvant RT should remain standard of care; organ preservation with induction chemotherapy and RT may be reasonable alternative for certain patients
Fu et al. 2016^49^	China	MA	Advanced laryngeal cancer	Total laryngectomy, nonsurgical organ‐preservation (chemotherapy, RT)	Local control, survival	Trend toward better overall, disease‐specific survival for total laryngectomy, but no clear difference in oncological outcomes; other factors (T‐stage, tumor size, lymph node metastasis, physical condition) also important indicators for treatment choices
Janssens et al. 2016^60^	Netherlands	RCT	Advanced laryngeal cancer	Accelerated RT with/without carbogen, nicotinamide	QoL	Good local tumor control, speech, swallowing function with accelerated RT; one‐quarter of patients have long‐term dry mouth, sticky saliva, taste/smell changes
Bottomley et al. 2014^43^	Belgium	RCT	Advanced laryngeal cancer	Sequential induction, alternating chemotherapy/RT	QoL	Trend toward worse scores with alternating chemoradiotherapy but very few significant differences; most patients' health‐related QoL scores returned to baseline after therapy
Shapira et al. 2022^81^	Israel	MA	Early glottic cancer	Open, trans‐oral salvage partial laryngectomy	Laryngectomy‐free survival	High survival rates for open (90.4%) and trans‐oral (78.6%) techniques in well‐selected patients after RT failure
Campo et al. 2022^45^	Italy	SR	Early glottic cancer	Open partial laryngectomy, total laryngectomy	Survival	High success of open partial laryngectomy for selected pT3 cases, accurate selection of cases amenable to conservative surgery important
Kachhwaha et al. 2021^62^	India	RCT	Early glottic cancer	Hypofractionated, conventional RT	Survival	No difference in overall survival, hypofractionated regimen provides better local control, symptomatic relief with shorter treatment time
Feng et al. 2011^48^	China	MA	Early glottic cancer	Laser surgery, RT	Oncological outcomes	No difference in cure rate, inconclusive voice preservation results
Rodrigo et al. 2019^79^	Spain, Germany, Belgium, Slovenia, Italy, USA	SR	Early glottic cancer	Transoral laser microsurgery	Survival	5‐year disease‐specific survival 95%, overall survival 68%, laryngectomy‐free survival 88%; procedure safe and effective for cases with few complications, good local control (>85%) and disease‐specific survival (>90%)
Mo et al. 2017^35^	China	MA	Early glottic cancer	Transoral laser microsurgery, RT	Oncological outcomes, QoL	Better overall survival and laryngeal preservation with laser surgery than with RT, no difference in local control
van Loon et al. 2012^92^	Netherlands	SR	Early glottic cancer	Laser surgery, RT	Functional outcomes, QoL	Only voice, QOL outcomes reported; heterogeneity of outcome measures prevented data pooling; uncertainty about tumor comparability (depth, extent), small samples, poor reporting hindered interpretation
Pakkanen et al. 2022^75^	Finland	RCT	Early glottic cancer	Transoral laser microsurgery, RT	Survival, larynx preservation	Similar results for both treatment modalities
Gioacchini et al. 2017^50^	Italy	SR	Early glottic cancer	Transoral laser microsurgery, RT, open partial laryngectomy	Survival	Better disease‐free survival with RT (87%), open partial laryngectomy (83%) than with transoral laser microsurgery (77%)
Reinhardt et al. 2022^77^	Switzerland	RCT	Early glottic cancer	Single vocal cord irradiation, transoral CO₂‐laser microsurgical cordectomy	Functional, oncological outcomes	‐
Huang et al. 2022^58^	China	MA	Early glottic cancer	Laser surgery, RT	Survival	Better survival of T1a N0 M0 glottic cancer with laser surgery
Huang et al. 2017^55^	China	MA	Early glottic cancer	Laser surgery, RT	Oncological outcomes	Increased larynx preservation with laser surgery, no difference in local control, overall survival, or disease‐specific survival
Zhou et al. 2021^94^	China	MA	Early glottic cancer	Transoral laser microsurgery with/without anterior commissure involvement	Survival	More local recurrence, less laryngeal preservation likely with anterior commissure involvement, no difference in 5‐year overall survival
Warner et al. 2014^29^	United Kingdom	SR	Early glottic cancer	RT, open surgery, endolaryngeal surgery with/without laser	Survival	No difference in 5‐year survival after RT and surgery (91.7% and 100% for T1 tumors, 88.8% and 97.4% for T2 tumors); 5‐year disease‐free survival after RT and surgery 71.1% and 100.0% for T1 tumors, 60.1% and 78.7% for T2 tumors
Tulli et al. 2020^90^	Italy	MA	Early glottic cancer	Surgery with anterior commissure involvement	Local control	Anterior commissure involvement negative prognostic factor for local control of T1 tumors at 5 years, needs to be considered in T staging of glottic tumors
Warner et al. 2017^30^	United Kingdom	SR	Early glottic cancer	Transoral laser microsurgery, external beam RT	Local control	5‐year local control similar (weighted averages, 75.81% for RT, 77.26% for microsurgery)
Benson et al. 2020^41^	India	MA	Early glottic cancer	Moderately hypofractionated RT	Local control, survival	Significantly improved local control vs. conventional fractionation, no impact on overall survival
Campo et al. 2021^46^	Italy	SR	Early glottic cancer	CO₂ transoral laser microsurgery, RT, open partial laryngectomy	Survival	Better local control at 5 years posttreatment with open partial laryngectomy (94.4%), no difference between RT (75.6%) and laser surgery (75.4%); better laryngeal preservation with primary open partial laryngectomy (95.8%) and laser surgery (86.9%) than with RT (82.4%) primary treatment
Huang et al. 2017^57^	China	MA	Early glottic cancer	Laser surgery, RT	Larynx preservation, local control, survival	Better larynx preservation with RT for T1a tumors, no difference in overall or disease‐ specific survival
Abdurehim et al. 2012^37^	China	MA	Early glottic cancer	Transoral laser surgery, RT	Oncological, functional outcomes	No difference in local control, overall survival, disease‐specific survival, posttreatment voice quality; better larynx preservation with laser surgery as initial treatment
Hendriksma et al. 2018^53^	Netherlands	SR	Early glottic cancer	Transoral CO₂ laser microsurgery, RT	Functional outcomes	Better laryngeal preservation with microsurgery for T2 tumors (88.8% vs. 79.0%); differentiation of tumors with normal (T2a), impaired (T2b) mobility important because the latter have poorer prognosis with microsurgery and RT; with adequate staging and treatment, anterior commissure involvement does not compromise oncological outcomes
Yoo et al. 2014^31^	Canada	SR	Early glottic cancer	Endolaryngeal surgery with/without laser, RT	Local control, survival	No difference in likelihood of local control, overall survival; less measurable voice perturbation with RT, no difference in patient perception; initial surgical treatment may increase likelihood of laryngeal preservation
Greulich et al. 2015^51^	USA	MA	Early glottic cancer	Transoral laser microsurgery, RT	Voice outcomes	No difference in VHI scores for T1 tumors, suggesting no clinically significant difference in functional voice outcomes
Vaculik et al. 2019^91^	Canada	MA	Early glottic cancer	CO₂ transoral laser microsurgery, RT	Oncological outcomes	Better overall survival, disease‐specific survival, laryngeal preservation with microsurgery
O'Hara et al. 2013^74^	United Kingdom	SR	Early glottic cancer	Transoral laser surgery, RT	Local control	3‐year local control rates with laser surgery and RT 88.9% and 89.3% for T1a tumors, 76.8% and 86.2% for T1b tumors
Bahig et al. 2021^40^	Canada, USA	RCT	Early glottic cancer	Vocal cord–only, complete laryngeal radiation	Voice outcomes	‐
Huang et al. 2017^56^	China	MA	Early glottic cancer	Laser surgery, RT	Voice outcomes	RT increased maximum phonation time, decreased fundamental frequency; no difference in VHI score, jitter, shimmer, or airflow rate
Huang et al. 2017^59^	China	MA	Early glottic cancer	Laser surgery	Voice outcomes	Reduced postoperative VHI, GRABS scores; improved overall postoperative vocal‐cord function and QoL, but not early postoperative vocal‐cord function or physiology
Sapienza et al. 2019^80^	Brazil, USA, Japan	MA	Early glottic cancer	Altered, conventional fractionation RT	Local control	Hypofractionation, hyperfractionation improved local control of T1 tumors and with anterior commissure involvement, but benefit may not persist for T2 tumors (consider alternative strategies)
Guimarães et al. 2018^52^	Brazil	MA	Early glottic cancer	Transoral laser surgery, RT	Oncological, functional outcomes	Better overall survival, disease‐specific survival, laryngeal preservation with laser surgery, no difference in local control
Kodaira et al. 2018^64^	Japan	RCT	Early glottic cancer	Accelerated‐, standard‐fractionation RT	Survival	No difference in 3‐year overall survival
Aaltonen et al. 2014^36^	Finland	RCT	Early glottic cancer	CO₂ laser surgery, external beam RT	Voice outcomes	Similar overall voice quality; RT may be treatment of choice for patients with demanding voice quality requirements
Nasef et al. 2016^72^	Egypt	RCT	Early glottic cancer	Transoral laser microsurgery, external vertical hemilaryngectomy	Functional outcomes	Better overall postoperative outcome with microsurgery, with shorter hospital stays, less need for tracheostomy, nasogastric tube, ICU admission
She et al. 2015^82^	China	MA	Early glottic cancer	CO₂ laser surgery	Oncological outcomes	Postoperative local recurrence rate related to anterior commissure involvement
Zhang et al. 2018^93^	China	RCT	Early glottic cancer	CO₂ laser microsurgery, low‐temperature plasma radiofrequency ablation	Voice outcomes	Both treatments effective for T1a tumors; potential advantages of radiofrequency ablation for voice function
Trotti et al. 2014^89^	Canada, USA	RCT	Early glottic cancer	Hyperfractionation, conventional fractionation RT	Local control	Nonsignificantly better 5‐year local control with hyperfractionation for T2 tumors
Higgins 2011^54^	Canada	MA	Early glottic cancer	Transoral CO₂ laser excision, external beam RT	Local control, voice outcomes	No difference in local control, laryngectomy‐free survival, or voice quality
Al Afif et al. 2022^38^	Canada	RCT	Early glottic cancer	Hyaluronic acid injection during transoral laser microsurgery	Voice outcomes	No significant impact on subjective, objective voice outcomes
Qu et al. 2012^76^	China	MA	Early glottic cancer	External radiation, transoral laser surgery	‐	Transoral laser surgery much less expensive, could be completed in the clinic
Moon et al. 2014^71^	Republic of Korea	RCT	Early glottic cancer	Hypofractionation, conventional fractionation RT	Local control, survival	Hypofractionation RT not inferior, similar toxicity profile, potentially better local control, shortened overall treatment time for T1–2 tumors
Lahav et al. 2020^65^	Israel	RCT	Early glottic cancer	CO₂ laser cordectomy, KTP laser surgery	Oncological, functional outcomes	KTP ablation has similar curative outcome, potentially better preservation of vocal fold architecture and function; clinical significance of findings unclear
Thomas et al. 2012^88^	United Kingdom	SR	Early laryngeal cancer	Open partial laryngectomy	Local control, survival	89.8% local control at 24 months, 79.7% overall survival, 84.8% disease free survival (*n* = 5061)
Ding & Wang 2019^47^	China	MA	Glottic cancer	Laser surgery, RT	Local control, survival	Better laryngeal preservation, overall survival with laser surgery, no difference in local control, recurrence, or disease‐specific survival
Boyle & Jones 2022^44^	United Kingdom	SR	Early laryngeal cancer	Endoscopic laser surgery, external beam RT	Functional outcomes, QoL	No clear advantage of either treatment; prospective studies with standardized assessment needed for valid comparison
Janssens et al. 2012^61^	Netherlands	RCT	Laryngeal cancer	Accelerated RT with/without carbogen inhalation, nicotinamide	Local control, larynx preservation, toxicity, survival	Better 5‐year regional control with carbogen inhalation and nicotinamide, equivalent toxicity
Strieth et al. 2019^85^	Germany	RCT	Early laryngeal cancer	Transoral microsurgery with microvessel‐ablative KTP laser, gold‐standard cutting CO₂ laser	Voice outcomes	Significantly reduced VHI scores, no relapse with KTP laser; one recurrence within 6 months with CO₂ laser
Sjogren et al. 2022^84^	Europe	SR	Glottic cancer	CO₂ transoral laser microsurgery	Voice outcomes	VHI scores increased gradually (range 14.2–21.5) but similar for all cordectomy types, dysphonia grade increased gradually with increasing resection depth (range 1.0–1.9), maximum phonation time decreased gradually (range 15.2–7.2)
Lyhne et al. 2015^68^	Denmark	RCT	Glottic cancer	Moderately accelerated, conventional fractionated RT	Local control	Moderately accelerated radiotherapy improved locoregional of glottic SCC

Abbreviations: Bcl‐xL, B‐cell lymphoma‐extra large; ICU, intensive care unit; KTP, potassium titanyl phosphate; MA, meta‐analysis; QoL, quality of life; RCT, randomized controlled trial; RT, radiation therapy; SCC, squamous cell carcinoma; SR, systematic review; VHI, Vocal Handicap Index.

## 4 DISCUSSION

The clinical landscape of glottic cancer has changed over the years, and, as shown by this review, new treatment modalities, mainly for organ function preservation, are being assessed. The NCCN guidelines, based on the most recent evidence generated throughout the world, provide recommendations that support clinicians' and patients' decision making. This study provides an overview of changes made to the guidelines and current recommendations for glottic cancer treatment. Over the study period, clinical stage–specific surgical principles and non‐surgical therapy for glottic cancer evolved in response to the development of new techniques, human and technological resources, and, mainly, a greater understanding of therapeutic modalities and corresponding survival rates. The intention to preserve the larynx and obtain better locoregional control and survival led to the performance of several studies of different clinical stages, mainly early, of glottic cancer (Figure 2, Table 3).

According to the NCCN categories of evidence and consensus, all recommendations appearing in the NCCN guidelines are category 2A (lower‐level evidence, uniform NCCN consensus) unless otherwise indicated. An example of a category 1 recommendation (high‐level evidence, uniform NCCN consensus) is that for the concurrent administration of ST and RT in the presence of extranodal extension of T1–T2 N0 and select T3 N0 cases. The category of evidence for induction chemotherapy recommendations for advanced glottic cancer changed considerably over the period of this study, from 2B (lower‐level evidence, nonuniform NCCN consensus) and 3 (any level of evidence, major disagreement) to 2A. These changes are broadly supported by evidence linking laryngeal preservation to clinical outcomes, and they reflect the increasing clinical relevance of such non‐surgical strategies.^9^, ^49^

RT and surgery (including endoscopic resection or open partial laryngectomies) remain the two main recommendations for early‐stage glottic cancer in the NCCN guidelines. Advances in surgical techniques with different laser modalities and robotic‐assisted transoral surgery were observed, however, not only surgical techniques improved, as RT is using intensity‐modulated radiation to precisely select normal tissues and targets for treatment, providing curative treatment with less toxicities.^44^, ^58^, ^75^, ^77^ As T1–T2 glottic cancer survival outcomes rates of laryngeal preservation are good and similar following laser surgery and RT,^6^ recent studies have focused on the improvement of functional voice outcomes.^5^, ^85^ Relative to transoral laser microsurgery using a CO₂ laser, that performed with an angiolytic potassium titanyl phosphate laser for early‐stage glottic cancer yielded significantly better functional voice results with adequate oncological safety, and the researchers have encouraged further studies with larger samples to inform the selection of the best surgical laser technique for patients with this disease.^85^ Other therapeutic modalities for early‐stage glottic cancer that have been assessed include intraoperative hyaluronic acid injection during transoral laser microsurgery, which had no significant effect on survival,^38^ and altered (hypo‐ and hyper‐) fractionation RT, which was found to result in fewer local failure events than did conventional RT.^80^ The selection of the appropriate treatment modality is individualized by assessing clinicopathological factors, patient characteristics, as well as preferences and expectations and, finally, the availability of human and technological resources, however, the larynx preservation approaches have become the standard of care for early‐stage glottic cancer.

Induction chemotherapy and clinical trials were the pathways added over time as treatment options for primary and neck in advanced glottic cancer. In this sense, the two previous modalities along with concomitant ST/RT or RT alone in patients whose performance status is not sufficient to tolerate this treatment, and surgery with or without adjuvant therapy, are the pathways provided by the last algorithm (2022) of the NCCN guideline. Although RCTs with a robust analysis between surgical and non‐surgical therapy comparing oncologic and functional outcomes in a homogeneous clinicopathological population are needed, retrospective studies performed in a population‐based database analyzing surgery with or without adjuvant treatment versus chemoradiation found superior oncological outcomes in those surgically treated.^8^, ^33^ Despite the controversies in the oncological results between studies comparing surgical and non‐surgical modalities,^13^, ^67^, ^69^, ^87^ it is noteworthy that induction chemotherapy, which has been gaining more evidence in recent years, is an excellent option for those patients with advanced cancer who are not candidates for surgery or those patients who, after careful evaluation by a multidisciplinary team, the decision to larynx preservation with dysfunction‐free survival through non‐surgical protocols is chosen according to good survival prognosis and patients' QoL. A current study on induction chemotherapy assessing other alternative agents with bioselection and two cycles with platinum, 5‐fluorouracil plus docetaxel, and a B‐cell lymphoma 2 protein inhibitor to increase the organ preservation rate have shown that the non‐surgical approach had better tolerability but did not improve oncological outcomes.^86^

Based on the literature search strategy outlined in the NCCN guidelines,^95^ we conducted a PubMed database search to identify key literature in the field of glottic cancer treatment. The largest proportions of identified studies were conducted in Asian and European countries, consistent with the references cited in the NCCN guidelines. Notably, although the NCCN guidelines serve as a reference in American countries, no study cited therein was conducted in Latin America. Thus, the inclusion of data from low‐ and middle‐income American countries with different human and technological resources available in future NCCN evidence reviews would be particularly important for the comprehensive assessment of differences in treatment protocols and clinical outcomes.

In general, the changes made to the NCCN glottic cancer treatment guidelines have been consistent with published research results, especially with the accumulation of clinical data for new techniques such as transoral laser microsurgery and induction chemotherapy.^5^, ^9^, ^35^ The guidelines support decision making about glottic cancer treatment that is individualized and focused on functional outcomes and patients' QoL.

## AUTHOR CONTRIBUTIONS

*Conceptualization*: Lady Paola Aristizabal Arboleda, Aline Borburema Neves, Maria Paula Curado, Luiz Paulo Kowalski; Methodology, Lady Paola Aristizabal Arboleda, Aline Borburema Neves. *Formal Analysis*: Letícia Miliano Candelária, Matheus Ferraz Borges, Gisele Aparecida Fernandes. *Writing—original draft*: Lady Paola Aristizabal Arboleda. Writing—review & editing: Hugo Fontan Kohler, José Guilherme Vartanian, Genival Barbosa de Carvalho, Alan Roger Santos‐Silva, Luiz Paulo Kowalski, Maria Paula Curado. *Visualization*: All authors. *Supervision*: Alan Roger Santos‐Silva, Luiz Paulo Kowalski, Paul Brennan, Maria Paula Curado. All authors approved the final manuscript as submitted and agree to be accountable for all aspects of the work.

## CONFLICT OF INTEREST STATEMENT

The authors declare that they have no conflict of interest related to this work.

## ETHICS STATEMENT

This is a literature review study, and no ethical approval is required according to the Ethics Committees of A.C.Camargo Cancer Center. This manuscript has been performed in an ethical and responsible way, with no research misconduct.

## Data Availability

Data sharing is not applicable to this article as no new data were created or analyzed in this study.

## APPENDIX A

**TABLE A1:** Search strategy and number of studies identified from Medline/PubMed.

Database search strategy search date (October 26, 2022)	Results	Filters applied	Total
(“Cancer of Larynx” OR “Larynx Cancer*” OR “Laryngeal Cancer” OR “Cancer of the Larynx” OR “glottis cancer” OR “glottic cancer” OR “glottic carcinoma”) AND (“conservative treatment”[MeSH Terms] OR “neoadjuvant therapy”[MeSH Terms] OR “Radiotherapy”[MeSH Terms] OR “induction chemotherapy”[MeSH Terms] OR “chemotherapy, adjuvant”[MeSH Terms] OR Surgery OR Surgical OR “robotic surgical procedures”[MeSH Terms] OR “Laryngectomy”[MeSH Terms] OR “endoscopic resection” OR “palliative care”[MeSH Terms] OR Treatment OR Management OR “Clinical Protocols”[Mesh] OR “Treatment Protocol*” OR “Antineoplastic Protocols”[Mesh] OR “Cancer Treatment Protocol*”)	7536	Results by year: 2011–2022 Results by article type: ‐Clinical trial ‐Controlled clinical trial ‐Meta‐analysis ‐Randomized controlled trial ‐Systematic review	260 results
